# Recommendations for accelerating open preprint peer review to improve the culture of science

**DOI:** 10.1371/journal.pbio.3002502

**Published:** 2024-02-29

**Authors:** Michele Avissar-Whiting, Frédérique Belliard, Stefano M. Bertozzi, Amy Brand, Katherine Brown, Géraldine Clément-Stoneham, Stephanie Dawson, Gautam Dey, Daniel Ecer, Scott C. Edmunds, Ashley Farley, Tara D. Fischer, Maryrose Franko, James S. Fraser, Kathryn Funk, Clarisse Ganier, Melissa Harrison, Anna Hatch, Haley Hazlett, Samantha Hindle, Daniel W. Hook, Phil Hurst, Sophien Kamoun, Robert Kiley, Michael M. Lacy, Marcel LaFlamme, Rebecca Lawrence, Thomas Lemberger, Maria Leptin, Elliott Lumb, Catriona J. MacCallum, Christopher Steven Marcum, Gabriele Marinello, Alex Mendonça, Sara Monaco, Kleber Neves, Damian Pattinson, Jessica K. Polka, Iratxe Puebla, Martyn Rittman, Stephen J. Royle, Daniela Saderi, Richard Sever, Kathleen Shearer, John E. Spiro, Bodo Stern, Dario Taraborelli, Ron Vale, Claudia G. Vasquez, Ludo Waltman, Fiona M. Watt, Zara Y. Weinberg, Mark Williams

**Affiliations:** 1 Office of the President, Howard Hughes Medical Institute, Chevy Chase, Maryland, United States of America; 2 TU Delft OPEN Publishing, Delft University of Technology—TU Delft Library, Delft, the Netherlands; 3 Department of Public Health, UC Berkeley School of Public Health, Berkeley, California, United States of America; 4 The MIT Press, MIT, Cambridge, Massachusetts, United States of America; 5 Development, The Company of Biologists, Cambridge, United Kingdom; 6 Medical Research Council—UKRI, London, United Kingdom; 7 ScienceOpen GmbH, ScienceOpen, Berlin, Germany; 8 Cell Biology and Biophysics, European Molecular Biology Laboratory, Heidelberg, Germany; 9 Technology, Sciety/eLife, Cambridge, United Kingdom; 10 GigaScience Press, GigaScience Press, Hong Kong, Hong Kong SAR; 11 Knowledge & Research Services, Bill & Melinda Gates Foundation, Seattle, Washington, United States of America; 12 Biochemistry Section, Surgical Neurology Branch, National Institute of Neurological Disorders and Stroke, National Institutes of Health, Bethesda, Maryland, United States of America; 13 Health Research Alliance, Swanton, Vermont, United States of America; 14 Bioengineering and Therapeutic Sciences, University of California San Francisco & ASAPbio, San Francisco, California, United States of America; 15 National Center for Biotechnology Information, National Library of Medicine, National Institutes of Health, Bethesda, Maryland, United States of America; 16 Centre for Gene Therapy and Regenerative Medicine, King’s College London, London, United Kingdom; 17 Literature Services, EMBL-EBI, Cambridge, United Kingdom; 18 The San Francisco Declaration on Research Assessment, Rockville, Maryland, United States of America; 19 PREreview, Portland, Oregon, United States of America; 20 Digital Science, London, United Kingdom; 21 Publishing Section, The Royal Society, London, United Kingdom; 22 The Sainsbury Laboratory, Norwich, United Kingdom; 23 cOAlition S, Guildford, United Kingdom; 24 The American Society for Cell Biology, Rockville, Maryland, United States of America; 25 Open Research, PLOS, San Francisco, California, United States of America; 26 F1000, London, United Kingdom; 27 Open Science Implementation, EMBO, Heidelberg, Germany; 28 President’s Office, European Research Council, Brussels, Belgium; 29 PeerRef, Pontefract, United Kingdom; 30 Open Science, Hindawi, London, United Kingdom; 31 Unaffiliated, Washington, DC, United States of America; 32 Qeios, London, United Kingdom; 33 SciELO Preprints, SciELO, São Paulo, Brazil; 34 Review Commons, EMBO, Heidelberg, Germany; 35 Science Program, Instituto Serrapilheira, Rio de Janeiro, Brazil; 36 eLife, Cambridge, United Kingdom; 37 ASAPbio, Somerville, Massachusetts, United States of America; 38 DataCite, Cambridge, United Kingdom; 39 Crossref, Oxford, United Kingdom; 40 Biomedical Sciences, University of Warwick, Coventry, United Kingdom; 41 Cold Spring Harbor Laboratory, New York, New York, United States of America; 42 COAR (Confederation of Open Access Repositories), Göttingen, Germany; 43 Simons Foundation, New York, New York, United States of America; 44 Chan Zuckerberg Initiative, Redwood City, California, United States of America; 45 Janelia Research Campus, HHMI, Ashburn, Virginia, United States of America; 46 Biochemistry Department, University of Washington, Seattle, United States of America; 47 Centre for Science and Technology Studies (CWTS), Leiden University, Leiden, the Netherlands; 48 EMBO, Heidelberg, Germany; 49 Biochemistry & Biophysics Department, University of California San Francisco, San Francisco, California, United States of America

## Abstract

Peer review is an important part of the scientific process, but traditional peer review at journals is coming under increased scrutiny for its inefficiency and lack of transparency. As preprints become more widely used and accepted, they raise the possibility of rethinking the peer-review process. Preprints are enabling new forms of peer review that have the potential to be more thorough, inclusive, and collegial than traditional journal peer review, and to thus fundamentally shift the culture of peer review toward constructive collaboration. In this Consensus View, we make a call to action to stakeholders in the community to accelerate the growing momentum of preprint sharing and provide recommendations to empower researchers to provide open and constructive peer review for preprints.

## Abstract 2

As preprints become more widely used and accepted, they raise the possibility of rethinking the peer-review process. This Consensus View issues a call to action to accelerate the growing momentum of preprint sharing and provides recommendations to empower researchers to provide open and constructive peer review for preprints.

## Introduction

Critical views (“reviews”) from independent researchers (“peers”) can identify conceptual, logical, or methodological gaps in scientific work. Peer review has thus become a key feature of the scientific process and is used in funding and evaluation [1,2], as well as to assess articles, which can help authors improve manuscripts and give readers (including the general public) increased confidence in the findings reported. Although many researchers are now comfortable making manuscripts publicly available as preprints before peer review, surveys report that 90% of researchers believe that peer review improves the quality of published work [3] and has enhanced the most recent paper they published [3,4].

Nevertheless, journal peer review faces many challenges [5]. It can be slow, inefficient, error-prone, inequitable, and unduly focused on providing advice to a journal editor to aid their decision. Despite requiring a huge time investment by the research community [6,7], peer review by 2 or 3 individual researchers cannot detect all of the problems in a study [8]. As a result, serious flaws may only come to light after journal publication, when a paper becomes visible to a broader group of experts. Meanwhile, a lack of transparency can mask errors and bias in the peer review process [9]. While over 500 journals now publish peer reviews alongside published articles [10], the majority do not [11,12], and peer reviews of rejected papers are almost never made public. This is a wasted opportunity to provide recognition for reviewers, additional contextual information that could help readers of an article to assess its merits, and the transparency necessary to study and improve the peer review process. In addition, reviewers may be charged with judging whether a paper is sufficiently exciting or “complete” for a particular journal. This can contribute to a tendency for reviewers to suggest additional experiments or analyses rather than providing advice focused solely on the work presented. As a result, life sciences articles are now often expected to include significantly more data than in previous decades [13,14], which creates an additional burden for authors and ultimately slows the dissemination of new scientific evidence.

The growing adoption of preprints (with the ratio of preprints to journal articles reaching 6.4% in 2020 across all disciplines [15]) offers an important opportunity to experiment with new approaches to peer review that could help address these issues. New approaches involving open peer review on preprints could also provide benefits to authors, reviewers, and readers. This Consensus View, which is the outcome of discussions at the Recognizing Preprint Peer Review workshop [16], provides recommendations to empower researchers to provide open and constructive peer review for preprints, and issues a call to action to stakeholders in the community to accelerate the growing momentum of preprint sharing.

## Methodology

The Recognizing Preprint Peer Review workshop took place on December 1–2, 2022 at the Howard Hughes Medical Institute’s Janelia Research Campus (Virginia, United States of America) and brought together representatives of funders, institutions, preprint servers, journals, indexers, infrastructure providers, and review services to discuss steps to drive community support and recognition for preprint peer review. Prior to the workshop, two Working Groups—one focused on funder, researcher, and institution perspectives and the other on journal and preprint review project perspectives—discussed preprint feedback and review and its potential uses as part of their respective processes. The Working Groups developed an initial definition of preprint feedback and preprint review, as well as recommendations for different stakeholders, which were presented and discussed further as part of the sessions at the workshop. Input from participants in the workshop was also collected via online polls.

The authors of this article are a subset of participants invited to the workshop and are primarily located in North America and Europe. Outside of gender balance, participant demographics were not representative of researchers in these regions, being skewed toward those from a white ethnic background and senior career stages. The ideas and recommendations offered in this article summarize those covered during the workshop, while reflecting the authors’ identities, backgrounds, values, and levels of engagement with the topics discussed.

### The state of preprint review

#### Defining preprint feedback and review

Feedback on preprints is not bound by the expectations of journal peer review. As a result, a variety of forms of preprint feedback have emerged. Indeed, automated services such as the Automated Screening Working Group and ScienceCast are processing and summarizing preprints. Some preprint servers, such as bioRxiv and medRxiv, highlight their outputs [17]. Nevertheless, we defined the scope of our meeting to include only human-generated feedback, ranging from minimal and informal approaches to in-depth formal peer reviews.

The diversity of human feedback creates a need to formalize the definition of preprint review. Based on input from the two Working Groups convened in advance of the Recognizing Preprint Peer Review workshop [18,19], the participants at the meeting defined preprint review as a subset of public preprint feedback that meets certain criteria. Box 1 distills the outcome of discussions about definitions of preprint review from the meeting.

**Box 1. Defining preprint feedback and review**

**Preprint feedback** is publicly available commentary on a preprint that is written by a human.

A **preprint review** is a specific type of preprint feedback that has:

- Discussion of the rigor and validity of the research.
- Reviewer competing interests declared and/or checked.
- Reviewer identity disclosed and/or verified, for example, by an editor or service coordinator, or ORCID login.

The categorization of “preprint feedback” and “preprint review” does not assign more or less value to comments falling in either category, as this depends on the use the reader makes of the feedback (for example, anonymous and unverified comments on PubPeer that call attention to image duplication are valuable to the community). Rather, it reflects the discussions among funders, institutions, and journal representatives noting that feedback that meets the criteria listed for preprint review is more likely to be incorporated in their evaluations, including the availability to verify the reviewer’s identity independently of whether this is publicly shared.

It was also clear in the discussions that this description of preprint review encompassed a minimal set of requirements. Additional points discussed included whether a minimum number of independent reviews should be required and whether the review process should result in an explicit recommendation or endorsement of the work (akin to an accept/reject recommendation for a journal). Whether the term “peer” should be part of the definition was also debated. Several participants indicated that an understanding of the reviewer’s expertise is necessary in order to establish whether they constitute a “peer,” and that this determination requires knowledge of the reviewer’s identity or a public description of their areas of expertise. Others noted that the term “peer” may be interpreted differently by different communities and that peer review can involve individuals who bring a valuable external perspective (e.g., patient reviews). With this in mind, we opted to leave the determination of whether or not the individual contributing the preprint review constitutes a “peer” to the user of that review.

### Adoption of preprint review

**Fig 1:**
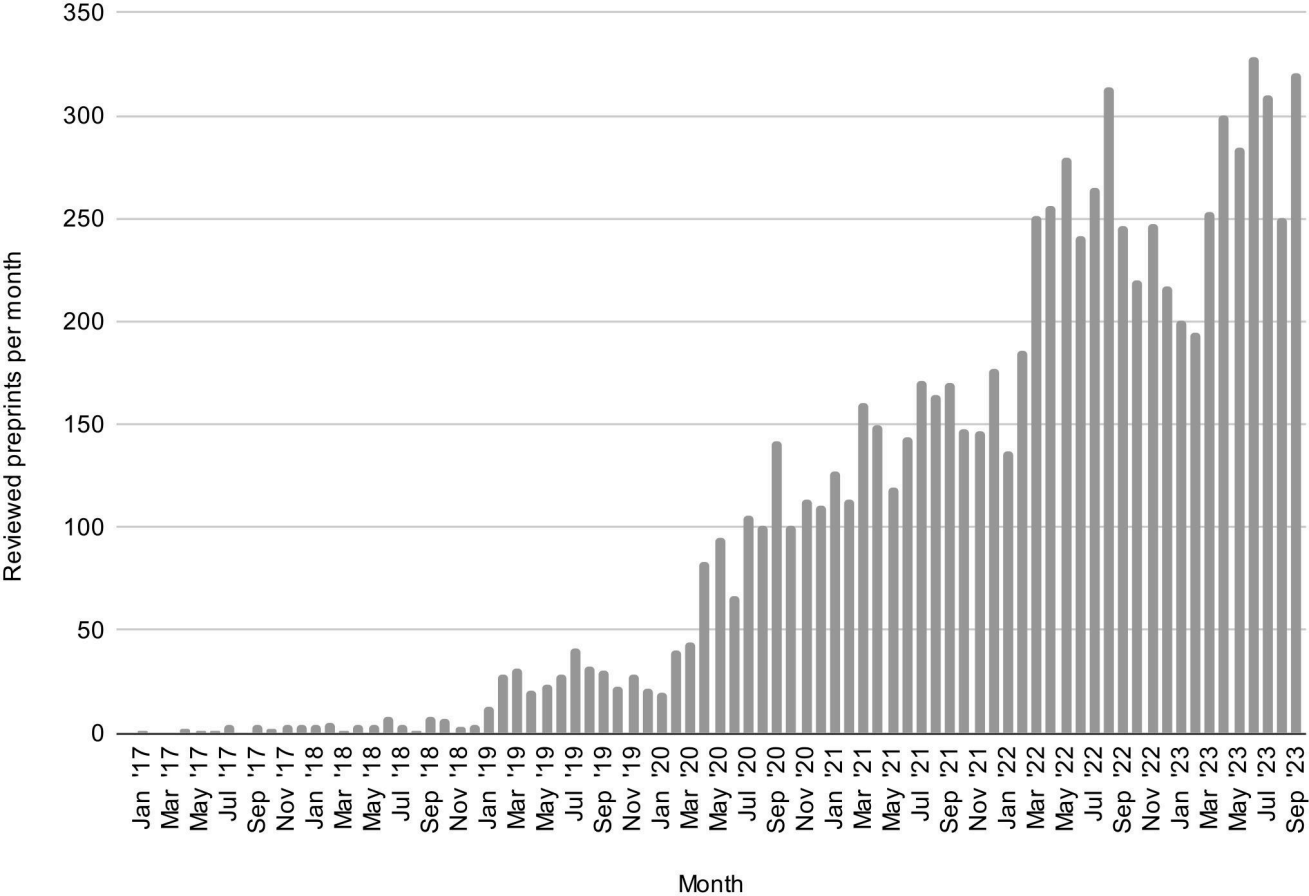
Estimating the growth of preprint review over time. Preprints evaluated per month on Sciety, excluding reviews conducted by automated tools (ScreenIT) and reviews by journals posted after publication of the journal version (source data available [20]). This chart includes data from the following services, regardless of which server the preprints they evaluate have been posted to: eLife, Review Commons, Arcadia Science, preLights, Rapid Reviews, PREreview, NCRC, Peer Community In (Evolutionary Biology, Ecology, Zoology, Animal Science, Neuroscience, Paleontology, Archaeology), PeerRef, Biophysics Colab, ASAPbio (and ASAPbio-SciELO) crowd review, Life Science Editors (including Foundation), and The Unjournal. Data have been collected and provided by Sciety. Reviews posted to comment sections of preprint servers are not included, and depending on the policies of individual services, some of the evaluations included in this chart may not meet our definition of preprint review.

Preprint reviews are being posted at an increasing rate (Fig 1). Multiple preprint review services (Box 2) whose outputs meet the above definition (including Review Commons, Peer Community In, PeerRef, PREreview, Qeios, and Rapid Reviews\Infectious Diseases) were represented at the workshop. They illustrate the diversity of approaches—from spontaneous posting of reviews by individuals to community-driven review platforms—that can be used to satisfy the above criteria.

**Box 2. Relevant preprint review services**

Preprint feedback and review sources (including communities that coordinate and post preprint evaluation prior to journal acceptance and platforms where evaluation may be hosted):

- Arcadia Science
- ASAPbio (and ASAPbio-SciELO) crowd review
- Biophysics Colab
- eLife
- Hypothesis
- Life Science Editors (including Foundation)
- Novel Coronavirus Research Compendium (NCRC)
- Peer Community In (Evolutionary Biology, Ecology, Zoology, Animal Science, Neuroscience, Paleontology, Archaeology)
- PeerRef
- preLights
- Preprint server commenting sections
- PREreview
- PubPeer
- Qeios
- Rapid Reviews\Infections Diseases
- Review Commons
- ScienceOpen
- The Unjournal

Frameworks and services for exchanging preprint review metadata:

- COAR Notify
- Crossref
- DataCite
- DocMaps
- Early Evidence Base
- Sciety

Prior to widespread adoption of preprinting in biology, some publishers had already implemented workflows that in many respects resemble preprint review. For example, Copernicus’s discussion journals encouraged community comments on manuscripts under peer review, and F1000 developed a model in which manuscripts are published first and then undergo open peer review (reviewers in the F1000 model assign report statuses that contribute to a threshold for passing peer review). As posting of preprints has become more common over the past decade, many journals have adopted policies that support preprinting and the publication of peer-review reports. Furthermore, new preprint review initiatives that decouple peer review from journals have emerged, including platforms such as PREreview and Peer Community In. More recently, eLife has introduced a new editorial model in which Reviewed Preprints are the primary output [21]. Review Commons has successfully implemented journal-agnostic preprint peer review in partnership with a growing consortium of journals. Furthermore, in 2022, preprints with reviews were recognized as satisfying the “peer-reviewed publication” eligibility criterion for EMBO Postdoctoral Fellowships [22], 9 funders committed to recognizing reviewed preprints in assessments [23], and cOAlition S stated that, for many of its funders, a paper that has “been subject to a journal-independent standard peer review process with an implicit or explicit validation” will be considered equivalent to a journal-reviewed article [24].

At the workshop, our discussions focused on the peer review itself, rather than on any decision or “validation” that follows from it. While some preprint review projects such as Review Commons deliberately avoid making editorial decisions (leaving this to the journal to which the reviews may be transferred), others provide endorsements or other shorthand signifiers of rigor and impact. Peer Community In, for example, only publishes reviews of articles that have been “endorsed” by their recommender (i.e., by the person coordinating the peer review for the preprint). Other services do not provide an accept/reject decision: Rapid Reviews\Infectious Diseases assigns scores to papers, and eLife’s new model deliberately moves away from accept/reject decisions, instead using a controlled vocabulary to express editorial judgments about the strength of the evidence and the significance of the findings in a summary statement published alongside the reviews. These varied approaches may yield different benefits for authors and readers. In cases where an explicit recommendation is not made, reviewers may feel liberated to focus on providing feedback for the author. By contrast, preprint review models that create a recommendation compress reviewers’ opinions into a concise and digestible message that can help readers sift through the scientific literature.

### Benefits of preprint review

When peer reviews of preprints are made publicly available, readers are able to see the reports alongside the article, evaluate the claims, and join the conversation. Interactions between authors, reviewers, and readers stimulated by public posting of reviews may surface perspectives from a larger and more diverse sample of the community, increasing the robustness of the assessment and providing further context. This can enable a wider audience, including non-specialists, to benefit from these insights and gain an understanding of how experts perceive the strengths and weaknesses of an article. Preprint review can also give readers more rapid access to peer-reviewed information because, unlike journal publication, reviews can be made available immediately rather than after multiple cycles of review and revisions (e.g., preprint reviews are posted a median of 46 days after the article [25], in contrast to an observed 199-day delay between preprinting and journal publication [26], or the 163 days from submission to journal publication seen elsewhere [27]).

Preprint review offers important benefits to trainees, who can learn good practices from reading public reviews. In addition, while early career researchers in certain disciplines may seldom be invited to peer review by journal editors, they can freely participate in many forms of preprint feedback and review, offering valuable perspectives; early career reviewers may be more attentive and have hands-on experience with new techniques that may be less familiar to senior reviewers. Some preprint review platforms (such as PREreview) offer structured templates that can help guide new reviewers [28]. Preprint review is already being incorporated into undergraduate and graduate courses on scientific publishing [29–31]. Furthermore, by focusing journal clubs on preprints rather than journal publications, participants can move beyond simply discussing a paper that is unlikely to change to producing reviews that will help authors and readers.

Preprint review can also benefit journals. For example, journals can use preprint reviews (as well as informal comments on preprints) to identify papers to invite for submission. In some cases, they may choose to reuse the reviews to expedite their own peer-review process, reducing the burden on the reviewer pool, and—when reviews are signed—providing useful leads to identify qualified peer reviewers for other papers.

In addition to these benefits, we believe that preprint review can promote a cultural shift in peer review. Reviewers can focus on the research as it stands, without having to comment on its fit for a particular journal. Open dialog may encourage reviewers to be more collegial and constructive. Authors could use the opportunity offered by preprint review to publicly respond to questions and concerns, thereby ensuring that their responses can be read by all. Finally, by making the comments of reviewers an integral element of scholarly discourse, peer review will increasingly be seen as a scholarly contribution in its own right.

### Challenges of preprint review

Despite the benefits noted above, preprint review is not without potential challenges. Participants raised a variety of concerns at the workshop, and we discussed how each can be addressed with thoughtful implementation of services and policies.

Preprint review services must address bias and non-collegial input, which can be serious problems in peer review [32]. In the most informal preprint feedback models, anyone may comment on a paper, and anonymous and pseudonymous feedback is permitted; thus, it becomes even more important to address the question of competing interests through transparent declarations or editorial oversight if these comments are to be treated as preprint reviews. Likewise, services that have editors or coordinators can promote constructive dialog through moderation of comments. Transparent disclosures about the nature of this moderation will help ensure readers use feedback appropriately. But for more open models that aim to minimize the impact of bad actors through community consensus, we need to nurture a culture in which norms of collegiality are established through training and community regulation [33].

Although the practice of posting preprints has been growing, only a minority of biomedical papers are posted as preprints (we estimate less than 10% [34]). Concerns about preprints differ across countries [35], with worries about scooping being more prominent among researchers in China than in the USA and Europe. Furthermore, perceived benefits of preprints vary, with one study finding that respondents in the Global South were more likely to strongly agree with benefits related to preprint visibility than those in the Global North [36]. Currently, preprint adoption varies across disciplines and geographies [37], with the highest rates of preprinting found in the USA and UK. Thus, not all communities may be ready to embrace preprint reviewing yet, and disciplinary and/or geographical differences in participation may also arise in the context of preprint review. While we should be mindful of supporting adoption in a manner that fosters inclusive participation, this should not deter progress on preprint review among those who are ready. At the same time, policies or guidance for preprint review must avoid undermining the value of the preprints themselves. Many funders and institutions recognize preprints cited in CVs and job or grant applications as research outputs alongside journal articles [38]. Recognition for preprint review must build on recognition for preprints. Preprint review can support and enrich evaluation of these articles, but the presence of such reviews does not itself signal the quality of the work.

Not all preprint authors will feel comfortable actively soliciting reviews of their papers. Those who submit to review services are both willing to risk participation in nontraditional publication models and are comfortable with public critique of their work. Moreover, reviewers and editors may be more willing to perform preprint review for authors within their existing networks, potentially reinforcing Matthew effects (i.e., benefits accrue to those who are already privileged) [39]. On the other hand, preprints lower barriers to sharing: many preprints are never published in a journal, and this fraction varies from approximately 20% of preprints from researchers in high-income countries to approximately 40% of preprints from researchers in low-income countries, and is correlated with funding disclosures [26]. This suggests that preprints enable the release of scientific outputs that would not otherwise be shared. Free or low-cost reviewing approaches built on top of preprints can make peer review more accessible to authors who lack funding or stable research environments.

If the fraction of biomedical papers posted as preprints is currently small, the fraction of preprints that have reviews is even smaller, and it is not equitably distributed. Less than 2% of preprints have accompanying reviews [20]. Preprint review services would need to scale massively to provide reviews for all the preprints that are currently posted. Platforms that organize preprint review as a service should be easy to use and able to scale so that any researcher can request or contribute to preprint review. Journal editors often report challenges in finding reviewers for manuscripts, so it will be important for preprint review services to expand their pools of potential reviewers to enable them to respond to growing demand. Participating in preprint review, and thereby developing a portfolio of public reviews, may be particularly attractive for early career researchers, who are often underrepresented in journal peer review. This should provide an opportunity for both preprint review services and journals to not only expand their pool of active reviewers but to also include a more diverse group of researchers in the peer-review process. Institutions that recognize preprint review should support preprint review services financially and encourage researchers to participate in organized preprint review. It is also essential that preprint reviews be visible and citable; we have proposed a citation format elsewhere [40].

Despite the rarity of preprint reviews, it is possible for authors to receive an overwhelming amount of feedback on their papers through social media or other channels, forcing them to prioritize responses to only those that are most useful or offered in good faith. An inundation of comments or reviews would also be difficult for readers to digest. Thus, tools to help manage information overload (such as searching, indexing, and summarization) will eventually be useful to help both authors and readers identify the reviews most relevant to them.

While services and frameworks such as DocMaps and COAR Notify are emerging, many indexing tools do not adequately connect the distributed network of reviews to preprints [41], which can make it difficult for researchers and other stakeholders to discover preprint reviews. A positive example is Europe PMC, which currently ingests DocMaps metadata from Sciety and Review Commons to facilitate accessibility and visibility of preprint reviews; users can filter their search of preprints to those with reviews. Europe PMC will be working on ingesting Crossref preprint review DOI metadata next. Sciety is an example of a platform that is dedicated to aggregating preprint review and feedback activity in one place, making it easier for researchers to read reviews and discover related reviewed preprints. Users can organize, comment on, and highlight preprints of interest, raising visibility and aiding the discovery of the research. We urge other databases to implement similar measures. Furthermore, we emphasize that preservation strategies are required to ensure that reviews remain accessible in the future.

We also encourage preprint servers to import or aggregate links to external preprint reviews, as is currently done by bioRxiv. In the absence of such integrations, readers may be inclined to post reviews via the commenting system of the preprint server. Such systems do not currently issue DOIs or any other form of persistent identifier. Ideally commenters posting reviews should also provide an authenticated ORCID, but this may create a barrier to entry, so tension between best practices for long-term discoverability and adoption exists. There is also the question of whether all comments warrant such a formal logging within the scientific record, and if this is not the case, how to distinguish between reviews and informal feedback.

### Recommendations for preprint review

We believe that all of the above challenges are surmountable, and that we now have the tools and community support needed to embrace preprint review. We recommend the following actions for stakeholders interested in participating in and promoting preprint review (Box 3).

**Box 3. Recommendations for participating in and promoting preprint review**

For individual researchers:

- Request reviews and feedback for the next preprint that you post by submitting to a preprint review service and/or include on the first page of your preprint an explicit invitation to review it publicly.
- Agree to review preprints when invited to do so.
- Review preprints following recommended good practices [30] and post your reviews as citable objects. These may be reviews requested by a journal editor or those you decide to write independently. Consider informing authors about your review ahead of posting and leave them time to provide a thoughtful response.
- Convert your lab or graduate program journal club to a preprint review club in which discussions are written up, shared with the preprint authors for feedback, and publicly posted [39].
- List preprint reviews on your CV or lab website to promote their visibility.

For funders, departments, and institutions:

- Consider preprints and their reviews in evaluations for funding, hiring, degree requirements, fellowship eligibility, tenure, and promotion. Make this consideration explicit on your website and in application instructions, for example, by adopting a CV format that enables listing preprints and their reviews (where the candidate is an author of a preprint) and reviews of preprints (where the candidate is a preprint reviewer).
- Allocate funding and support for preprint review services.
- Provide peer review training that incorporates publicly posting reviews on preprints.

For journals:

- Accept preprint reviews as transferred peer reviews to inform editorial decisions.
- Encourage or require preprint posting at submission.
- Partner with preprint review initiatives.
- Consider posting reviews on preprints prior to acceptance.
- Implement a written policy encouraging preprint reviews. Suggested text has been recommended by the Journals & Preprint Review Projects Working Group [23].
- Consider adopting a preprint review model for your journal.
- Implement preprint scooping-protection policies (examples: EMBO Press, PLOS, The Company of Biologists) to allow time for preprint review to proceed.

For preprint review services:

- Facilitate preprint reviews that meet the criteria in Box 1; invest additional editorial or technical resources into validating the identity of reviewers and addressing competing interests, as required.
- Create machine-readable metadata for preprint reviews, for example, by registering DOIs or providing an API.

For preprint servers, indexing, and search tools:

- Create links between preprints and preprint reviews in a human- and machine-readable fashion.
- Enable authors to solicit reviews at the time of submission of their work to a preprint server.

For journalists and other non-specialist readers:

- Seek preprint reviews to provide additional perspectives on research you cover or use.

Our recommendations encourage all researchers and readers to participate in preprint review, whether by requesting, partaking, funding, or considering it. Journals can contribute to creating an environment that supports preprint review by creating policies that welcome it and promote its reuse. Institutions and funders can incentivize preprint review by recognizing it as a form of scholarship in grant applications, reporting, hiring, graduation, and tenure and promotion policies. Furthermore, preprint review services, preprint servers, and indexing and search tools will have an important role in facilitating these actions by making preprint reviews more discoverable.

## Conclusion

Just 10 years ago, preprinting in many disciplines barely existed. Today, preprints are becoming more commonplace, are indexed by major bibliographic databases, and are encouraged (or even required) by many funders. Although preprint review is in its infancy, momentum is building rapidly, and we feel the potential benefits are already evident. Building on the growing enthusiasm within the community, the time is right to promote the growth of this practice so that scholarly publishing may become more constructive, equitable, and transparent.
